# Different Modes of Retrovirus Restriction by Human APOBEC3A and APOBEC3G *In Vivo*

**DOI:** 10.1371/journal.ppat.1004145

**Published:** 2014-05-22

**Authors:** Spyridon Stavrou, Daniel Crawford, Kristin Blouch, Edward P. Browne, Rahul M. Kohli, Susan R. Ross

**Affiliations:** 1 Department of Microbiology, Institute for Immunology and Abramson Cancer Center, Perelman School of Medicine, University of Pennsylvania, Philadelphia, Pennsylvania, United States of America; 2 Department of Medicine, Perelman School of Medicine, University of Pennsylvania, Philadelphia, Pennsylvania, United States of America; 3 Koch Institute for Integrative Cancer Research, Massachusetts Institute of Technology, Cambridge, Massachusetts, United States of America; King's College London School of Medicine, United Kingdom

## Abstract

The apolipoprotein B editing complex 3 (A3) cytidine deaminases are among the most highly evolutionarily selected retroviral restriction factors, both in terms of gene copy number and sequence diversity. Primate genomes encode seven *A3* genes, and while A3F and 3G are widely recognized as important in the restriction of HIV, the role of the other genes, particularly A3A, is not as clear. Indeed, since human cells can express multiple *A3* genes, and because of the lack of an experimentally tractable model, it is difficult to dissect the individual contribution of each gene to virus restriction *in vivo*. To overcome this problem, we generated human A3A and A3G transgenic mice on a mouse A3 knockout background. Using these mice, we demonstrate that both A3A and A3G restrict infection by murine retroviruses but by different mechanisms: A3G was packaged into virions and caused extensive deamination of the retrovirus genomes while A3A was not packaged and instead restricted infection when expressed in target cells. Additionally, we show that a murine leukemia virus engineered to express HIV Vif overcame the A3G-mediated restriction, thereby creating a novel model for studying the interaction between these proteins. We have thus developed an *in vivo* system for understanding how human A3 proteins use different modes of restriction, as well as a means for testing therapies that disrupt HIV Vif-A3G interactions.

## Author Summary

APOBEC3 genes are part of the host's arsenal against virus infections. Humans have 7 APOBEC3 genes and determining how each specifically functions to inhibit retroviruses like HIV is complicated, because all 7 can be produced in a given cell type or tissue. This is important, because some viruses make their own factors, such as the HIV Vif protein, that block the anti-viral activity of APOBEC3 proteins. Moreover, there is interest in developing anti-viral therapeutics that enhance the action of APOBEC3 proteins. To overcome this limitation, we made transgenic mice that express two of the human proteins, APOBEC3A and APOBEC3G in mice that do not express their own APOBEC3. These mice were able to effectively block infection by several mouse retroviruses. Moreover, we found that APOBEC3A and APOBEC3G used different mechanisms to block infection *in vivo*. These transgenic mice have the potential to increase our understanding of how the human proteins function to restrict virus infection *in vivo* and should be useful for the development of therapeutics that enhance APOBEC3 proteins' antiviral function.

## Introduction

Retroviruses are enveloped RNA viruses that infect many different species including humans. The constant “battle” between retroviruses and mammalian cells has resulted in the evolution of proteins that act as cellular restriction factors. These restriction factors provide defense against retroviruses by blocking various points of the retroviral life cycle within the cell [1], [2].

One such family of restriction factors is the apolipoprotein B editing complex 3 (A3) cellular cytidine deaminases (CDA). While *A3* genes are found in all mammals, their number differs from species to species. For example, humans have 7 *A3* genes (*A3A* to *A3H*) while mice have only one gene. All proteins in this family contain at least one CDA domain that deaminates carbon 4 of cytidine in single-stranded DNA, resulting in a uracil that causes G to A transitions in the opposing strand [3]. A3G was the first member of the family shown to restrict retroviruses. A3G expression is interferon-inducible and is packaged within HIV-1 virus particles. Packaged A3G leads to a substantial decrease in viral infectivity by causing a high frequency of G to A mutations in the coding strand due to deamination of minus strand reverse transcripts [4], [5]. The abundance of G to A mutations can lead to degradation of the retroviral DNA or the creation of nonfunctional proviruses [6]. A3G preferentially deaminates cytidines that are in a CC motif [7], [8]. Furthermore, A3G inhibits viral reverse transcription and blocks viral cDNA accumulation [9], [10]. This inhibition may be achieved by physical interaction between A3G and the viral reverse transcriptase [11].

Packaging of A3G into virions is counteracted by HIV Vif (viral infectivity factor) protein. In virus-producer cells, Vif binds to A3G as well other A3 family members, and recruits cellular E3 ubiquitin ligase complexes, leading to ubiquitination and subsequent proteasomal degradation, thereby preventing packaging of A3G into budding virions [12]–[14]. Lentiviral Vif proteins show strong species-specificity. For example, HIV-1 Vif counteracts human A3G but only certain simian A3G homologues [15], [16]; it also does not interact with mouse A3 [17].

Other members of the A3 family are believed to affect other exogenous viruses as well as endogenous retrovirus/retroelement movement within the genome. In particular, human A3A is a potent inhibitor of IAP and MusD and other retrotransposons such as LINE-1 and this inhibition is CDA-independent, at least in cultured cells [18]–[20]. A3A also inhibits adeno-associated virus replication, a nuclear-replicating parvovirus, via CDA-independent means [20]. In monocytes, A3A restricts HIV-1 infection and the decrease in A3A levels that occurs during monocyte-to-macrophage development is concomitant with increased susceptibility to HIV-1 infection [21]. A3A is not packaged into HIV virions and is thought to restrict infection by targeting incoming virus [22]–[24]. In contrast, A3A is packaged in human T-lymphotropic virus type-I virions and restricts infection, at least in transfected cells [25]. A3A preferentially deaminates cytidines that are in a TC motif [26].

Different A3 family members block infection by diverse retroviruses from different species, including HIV-2 [27], porcine endogenous retrovirus [28], [29], xenotropic, Friend (F-MLV) and Moloney murine leukemia virus (M-MLV) [30]–[32] and mouse mammary tumor virus (MMTV) [33]. Additionally, A3 proteins may restrict other virus families, including parvoviruses [20], [34], hepatitis B virus [35]–[37], papillomaviruses [38] and herpes simplex virus I [39]. Thus, it has been suggested that A3 proteins exist, at least in part, to prevent zoonotic transmission of viruses [40].

While much has been learned about A3 proteins and their roles in virus restriction, little is known about how they function *in vivo*. Only the murine *A3* gene has been studied *in vivo* by our group and others, using naturally occurring genetic variants of mouse *A3* and knockout mice infected with MMTV and MLV [31]–[33], [41]–[45]. Interestingly, while the murine protein is packaged into virions and has CDA activity, it restricts murine retrovirus infection largely by inhibiting reverse transcription [33], [46], [47]. Here, we show that transgenic mice expressing the human A3A or A3G proteins restrict murine retrovirus infection *in vivo* in disparate ways. A3G was packaged into virions *in vivo*, leading to the deamination of both MLV and MMTV viral genomes. In contrast, A3A was not packaged, and appeared to restrict infection in a largely CDA-independent manner. Finally, we show that Vif/A3G interactions can be studied in this *in vivo* model, thus providing a potentially useful system for the analysis of small molecule inhibitors of A3 proteins and Vif.

## Results

### Generation of mice expressing human A3A and A3G

One of the complications of studying different A3 proteins in humans is that more than one of the 7 genes can be expressed in a given cell type. In contrast, the mouse has only a single gene, and knockout mice are viable [33]. To study the role(s) of individual human A3 proteins *in vivo*, we used the chicken β-actin regulatory region to drive expression of myc-tagged A3A and A3G and created transgenic mice on a C57BL/6 background. Two independent transgenic strains each that transmitted the A3G or A3A transgene were obtained. Each of these strains was then back-crossed onto the A3 knockout background (also C57BL/6) to generate mice containing functional copies of only the human genes.

**Figure 1:**
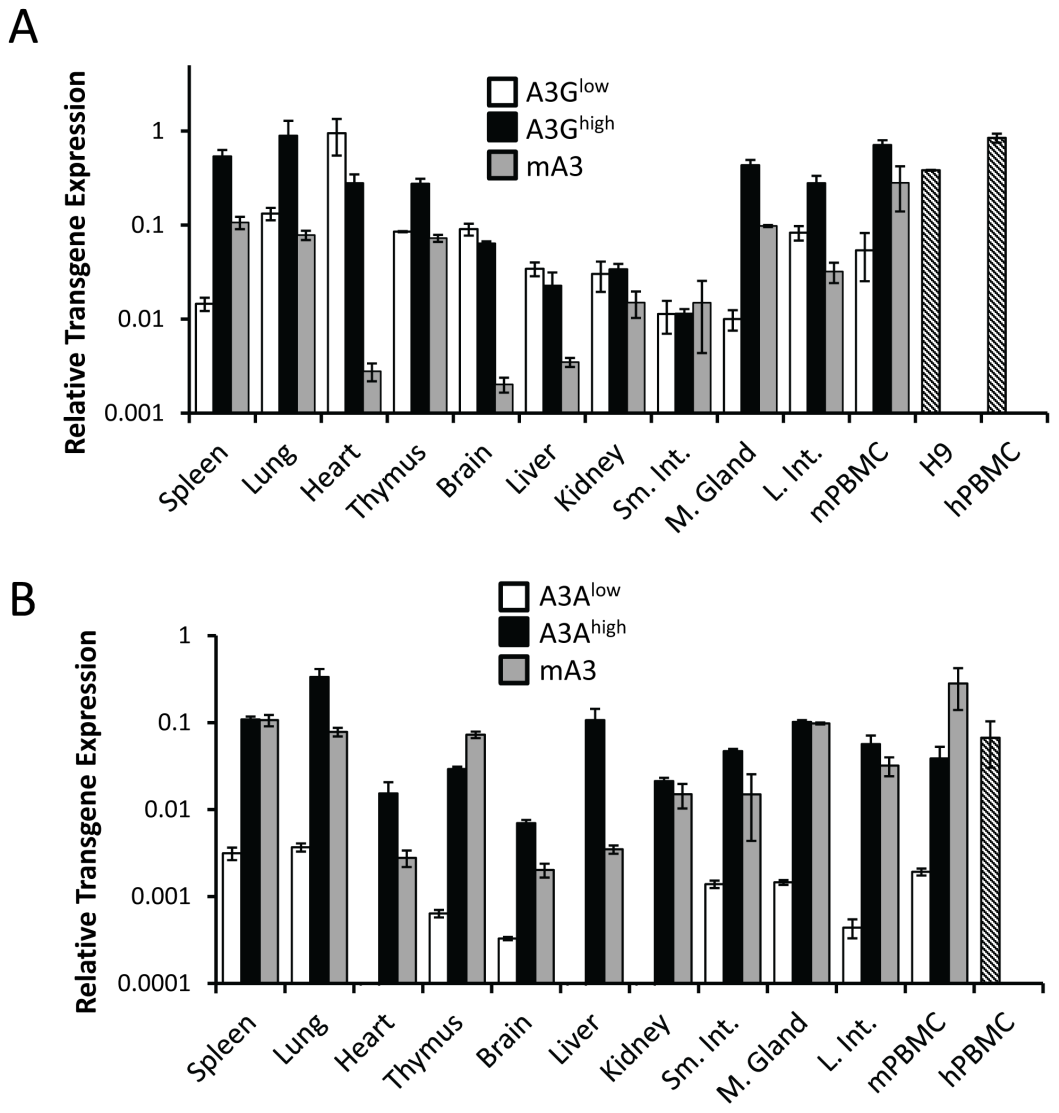
Expression of A3A and A3G transgenes. A) RT-qPCR analysis of RNA isolated from different tissues of the A3G^high^ and A3G^low^ strains. B) RT-qPCR analysis of RNA isolated from different tissues of the A3A^high^ and A3A^low^ strains. Shown for comparison for both graphs are the endogenous A3 levels in nontransgenic C57BL/6 mice (mA3), as well as A3A and A3G expression in human H9 cells and human PBMCs (average of 2 individuals). The mice used for this analysis were uninfected. Both panels are representative of 2 independent experiments with a different mouse of each genotype. Error bars denote standard deviation of technical replicates.

To determine the level of transgene expression, we first isolated RNA from different tissues, including peripheral blood mononuclear cells (PBMCs), and performed reverse-transcribed real-time quantitative PCR (RT-qPCR). RNA from human H9 cultured cells and human and C57BL/6 mouse PBMCs served as controls. For each transgene, there was one high- (A3G^high^, A3A^high^) and one low- (A3G^low^, A3A^low^) expressing strain, defined by their relative expression in lymphoid tissues. The A3G^high^ strain expressed higher levels of the transgene than the endogenous mouse gene in spleen and thymus, but similar A3G levels in mouse and human PBMCs, while the A3G^low^ strain expressed approximately 10-fold lower levels in these tissues (Figure 1A). In contrast, the A3A^high^ strain expressed similar or lower levels than mouse A3; there was also about 2-fold lower expression of A3A in mouse PBMCs than in human PBMCs (Figure 1B). The A3A^low^ strain had very low but detectable levels of expression in several tissues. Since the β-actin regulatory region was used, transgene expression was seen in many tissues and in several at levels higher than endogenous mouse A3 (e.g. heart, brain and liver) (Figure 1A and 1B). We also performed western blots on different tissues from the 4 different mouse strains, using antiserum that detects both A3A and A3G. The relative protein expression levels were similar to that seen at the RNA level (Figure S1A and S1B).

**Figure 2:**
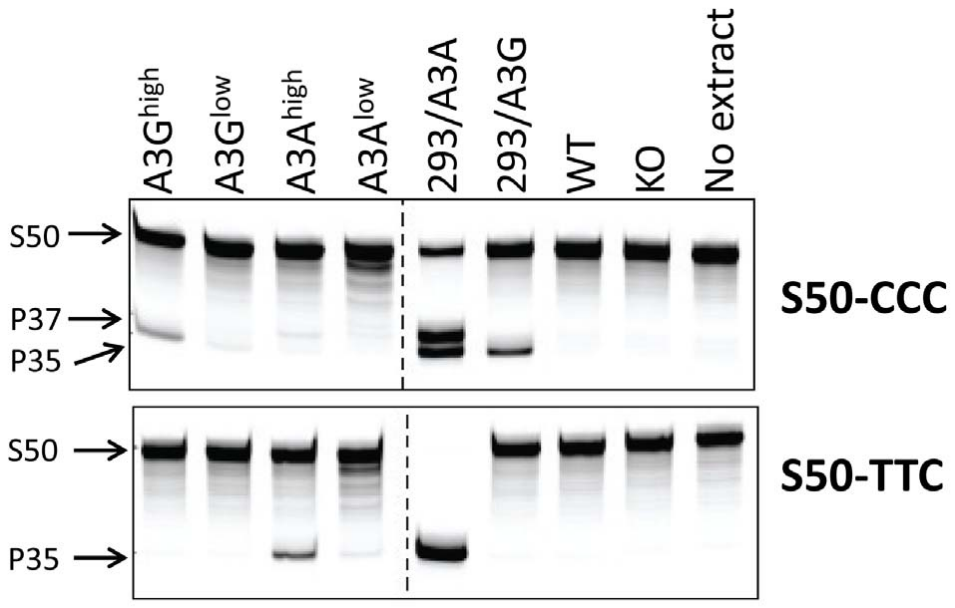
Cellular lysates from splenocytes derived from uninfected transgenic, wild type (BL/6) or mA3 knockout (KO) mice or from 293T cell lines over-expressing A3A or A3G were incubated with a 3′-fluorophore labeled 50-mer single-stranded oligonucleotide (S50) containing cytosine in the sequence context preferred by A3G (S50-CCC) or A3A (S50-TTC). Deamination was detected by uracil excision by UDG followed by fragmentation of the resulting abasic site by NaOH and heat, resulting in a 35-mer product (P35). High levels of activity in the 293/A3A samples result in deamination at multiple potential cytosines in the S50-CCC substrate (P37). This experiment was performed several times with the same lysates, with similar results.

We next determined if the *in vivo*-produced A3A and A3G proteins were functionally active. Extracts were prepared from primary splenocyte cultures and equal amounts (total protein concentration/volume) were incubated with FAM-labeled substrates containing the A3A- or A3G-preferred target sequence (S50-TTC and S50-CCC, respectively). As controls, we also performed these assays with extracts prepared from 293T cell lines transfected with A3A or A3G. Activity could be readily detected in transgenic mice expressing high levels of A3A or A3G. Further, in accord with the known specificity of the cytidine deaminases, extracts from the A3A^high^ mice deaminated the TTC- more efficiently than CCC-containing substrates, while those from A3G^high^ mice more efficiently deaminated the CCC substrate (Figure 2). For both A3A^low^ and A3G^ low^, trace amounts of activity were detectable with the preferred substrates, while no activity was detectable with either endogenous mA3 or from mA3 knockout splenocytes. No deaminase activity was detected with WT mouse extracts, perhaps because the mouse protein has lower overall activity or expression. These data show that the transgenic mice expressed catalytically active human deaminases in these heterologous cells.

### Human A3A and A3G restrict infection by murine retroviruses

A number of studies have demonstrated in transfected tissue culture cells that human A3G can restrict MLV and MMTV infection when it is packaged into virions [33], [48]–[50] whereas A3A, which is not packaged, did not restrict MLV [22], [51]. Both MLV and MMTV initially infect dendritic and other sentinel cells and then B and T lymphocytes during *in vivo* infection [32], [52]–[57]. To determine if the target sentinel/lymphoid cells expressed the transgenes, we sorted PBMCs from the transgenic mice into different populations and tested each for transgene RNA. In addition, we prepared bone marrow-derived dendritic cells (BMDCs) and macrophages from these mice. B and T cells, BMDCs and macrophages from the A3G^high^ and A3A^high^ strains expressed the transgene RNA (Figure S2). The A3G^low^ strain expressed the transgene in T and B cells and macrophages, but at lower levels in BMDCs, while the A3A^low^ strain had very low expression in all cell types. Interestingly, BMDCs showed the highest levels of expression of endogenous mouse A3. Thus, the transgenes were expressed in the appropriate MMTV and MLV target cell types, sentinel cells and lymphocytes.

**Figure 3:**
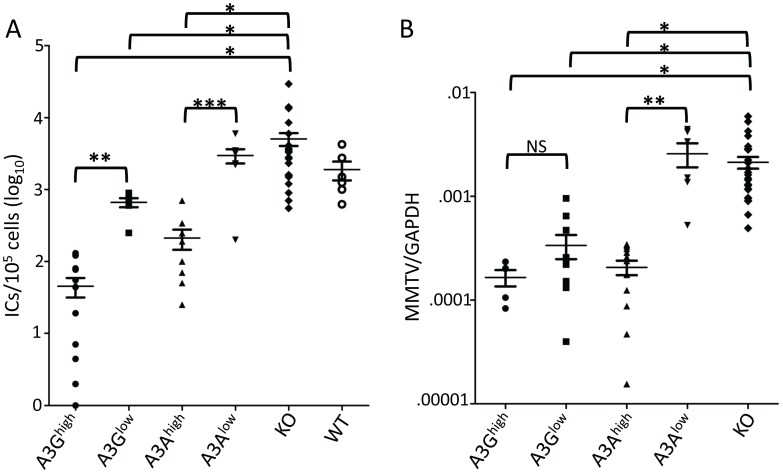
A3A and A3G restrict murine retrovirus infection *in vivo*. A) Newborn mice were infected with M-MLV and 16 days post-infection, virus titers in spleens were measured. Each point represents the titer obtained from an individual mouse; the average for each group is shown by a horizontal bar. The transgenic mice were derived from 2–3 litters each; the knockout mice are the littermates of the transgenic mice. N = 12 A3G^high^, 6 A3G^low^, 10 A3A^high^, 7 A3A^low^, 42 KO and 6 WT mice. B) Five day old mice were infected with MMTV and 3 weeks post-infection, DNA was isolated from spleens and subjected to RT-qPCR with MMTV-specific primers. Each point represents the proviral DNA levels measured in splenic DNA from an individual mouse; the average for each group is shown by a horizontal bar. The transgenic mice were derived from 2–3 litters each; the knockout mice are the littermates of the transgenic mice. N = 5 A3G^high^, 10 A3G^low^, 12 A3A^high^, 6 A3A^low^ and 24 KO mice. *, p≤0.0001, **, p≤.001, ***, p≤.01, NS, not significant (Mann-Whitney *t* test).

We next tested whether the human A3 proteins would function as anti-viral restriction factors *in vivo*. Newborn pups from each of the transgenic strains were infected with Moloney MLV (M-MLV) at 1 day after birth, and 16 days later, virus titers were obtained from splenocyte cultures of individual mice. The A3G^high^ mice had on average 2 logs lower viral titers than knockout or wild type mice while the A3G^low^ mice showed about 1 log lower infection (Figure 3A). While the A3A^high^ mice also had lower levels of virus, the A3A^low^ mice did not show statistically significant different levels of infection compared to knockout mice. Similar results were obtained with MMTV-infected mice inoculated with virus at 5 days and examined for infection at 21 days (Figure 3B). Thus, both A3A and 3G restrict murine retrovirus infection *in vivo*, even when expressed below the normal human endogenous levels.

### A3G but not A3A extensively deaminates MLV and MMTV DNA

It is well-recognized that A3G restricts HIV and other retroviruses by cytidine deamination, although it has been shown to also restrict infection by deaminase-independent means [15]. In contrast, mouse A3 restricts both MLV and MMTV by cytidine-deaminase-independent means, most likely by inhibiting reverse transcription [33], [46], [47], [58]. The role of A3A in restricting HIV infection is less clear. A3A is not packaged into HIV virions, and it has been suggested that it inhibits incoming virus in target myeloid cells [23], [24]. It is, however, believed to restrict endogenous retroelement retrotransposition and parvovirus replication by cytidine-deaminase-independent means [19], [20].

**Figure 4:**
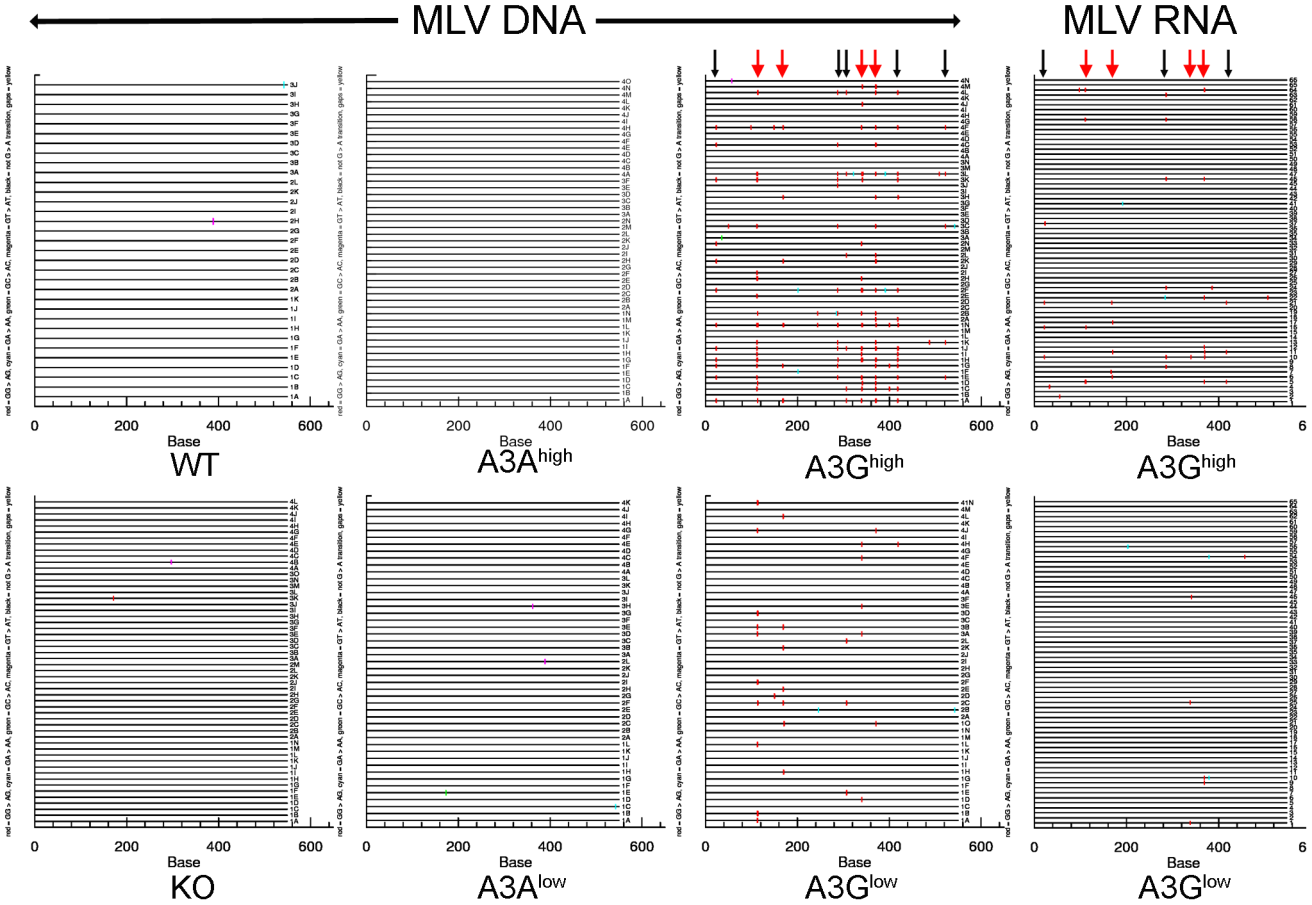
Deamination of M-MLV viral DNA and RNA in A3G transgenic mice. Splenic DNA or RNA from virions was isolated from the infected mice described in Figure 3 and cloned and sequenced. In most cases > 10 sequences from 4 different mice were analyzed, as indicated in the figure. For the viral RNA samples, the viruses from 5 animals were pooled for sequencing. Shown are the G to A changes in the sequences; other mutations are indicated in Table 1. Red  =  GG > AG, cyan  =  GA > AA, green  =  GC > AC and magenta  =  GT > AT transitions. Red arrows denote mutation hotspots seen in viruses isolated from A3G^high^ and A3G^low^ mice; black arrows denote hotspots identified only in A3G^high^ mice.

To determine the mechanism by which the human A3 proteins restricted MLV and MMTV infection *in vivo*, we isolated DNA from M-MLV- and MMTV-infected splenocytes, as well as RNA from M-MLV virions and sequenced a portion of the *env* gene of M-MLV and the *sag* gene of MMTV. These genes were chosen because of the ease of designing primers that did not amplify endogenous M-MLVs or MMTVs. Viral DNA isolated from both A3G transgenic mice showed evidence of heavy deamination and the extent of deamination was dependent on the level of transgene expression (Figure 4 and Figure S3; Table 1). While M-MLV viral RNA also showed evidence of deamination, particularly in the A3G^high^ strain, the level was much less than that seen in genomic DNA. This was mostly likely due to the selection of functional circulating viruses, in contrast to the integrated viral DNA, which when translated, demonstrated evidence of non-conserved amino acid changes and stop codons (not shown). Interestingly, both the viral DNA and RNA showed evidence of deamination hotspots (arrows in Figure 4; Table S1). These deamination hotspots were all in GG motifs.

**Table 1:** Mutation frequencies in MLV and MMTV proviruses in splenic DNA of infected A3A and A3G mice.

	Total sequenced	unique clones	# mice	# w/G to A	# w/o G to A	# G to A mutations	# other mutations	G to A kb	Other kb	GG	GA	GC	GT
**A3G^high^ M-MLV**	56	42	4	32	24	158	34	5.14	1.11	149	7	1	1
**A3G^low^ M-MLV**	47	28	4	23	24	35	48	1.33	1.82	33	2	0	0
**A3A^high^ M-MLV**	49	9	4	0	49	0	7	0.00	0.26	0	0	0	0
**A3A^low^ M-MLV**	47	17	4	4	43	4	38	0.16	1.47	0	1	1	2
**KO M-MLV**	54	24	4	2	52	2	34	0.07	1.15	1	0	0	1
**A3G^high^ MMTV**	57	40	4	29	28	68	59	1.77	1.54	58	8	1	1
**A3G^low^ MMTV**	52	31	4	19	33	27	58	0.77	1.66	14	4	8	1
**A3A^high^ MMTV**	52	29	4	6	46	12	62	0.34	1.77	1	10	1	0
**A3A^low^ MMTV**	41	20	3	4	37	4	46	0.14	1.67	2	1	1	0
**KO MMTV**	40	20	3	3	37	3	64	0.10	2.16	1	2	0	0
**A3G^high^ F-MLV**	41	25	3	27	14	116	43	4.83	1.79	113	2	1	0
**A3G^high^ F-MLV-vif**	53	23	4	6	47	12	71	0.39	2.29	11	0	0	1
**A3G^low^ F-MLV**	40	18	3	18	22	27	19	1.15	0.81	21	1	2	3
**A3G^low^ F-MLV-vif**	41	16	3	1	40	2	28	0.08	1.16	1	1	0	0
**KO F-MLV**	42	11	3	3	39	7	37	0.28	1.50	5	1	1	0
**KO F-MLV-vif**	42	42	3	1	41	1	67	0.04	2.72	1	0	0	0

Analysis was performed on the cloned DNA represented in Figure 4 (3–4 mice/group; 10–15 sequences/mouse). Unique clones were distinguished by their mutation pattern. M-MLV has a total of 47 GG and 33 GA motifs in the 549bp target sequence, MMTV has 34 GGs and 56 GAs in 673bp and F-MLV has 58 GG and 29 GA motifs in 586bp.

The results were similar for MMTV proviral DNA in spleen (Figure S3 and Table 1). MMTV proviral DNA was most heavily deaminated in the A3G^high^ strain, followed by the A3G^low^ strain although to a lesser extent than that seen in M-MLV; in both cases deamination preferentially occurred in GG motifs and there were also deamination hotspots (Table S1). Interestingly, in contrast to what was seen with M-MLV, there was very low but detectable deamination in the A3A^high^ strain (Table 1) at the GA consensus motif.

### A3G but not A3A is packaged in murine retroviruses

**Figure 5:**
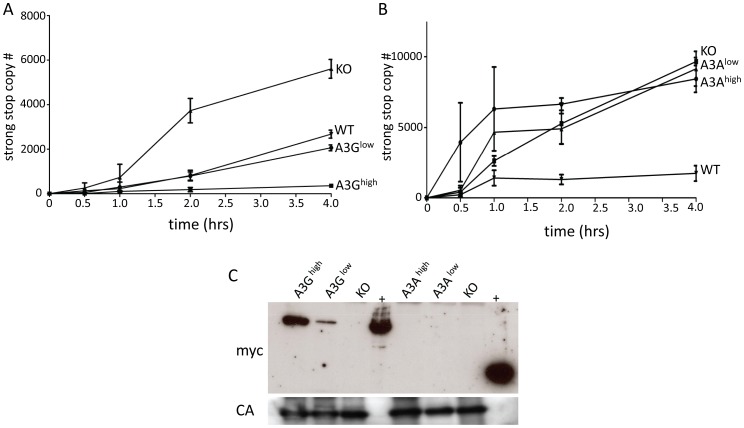
A3G but not A3A is packaged into M-MLV and inhibits reverse transcription. A) and B) Virions were isolated from the spleens of A3G (A), A3A (B), wild type and KO mice and EnRT assays were performed. Shown is the average of 3 independent experiments using different virus preparations; error bars show standard deviation. C) Western blot analysis of M-MLV virions from the transgenic mice, using anti-myc antisera (top panel). The blots were stripped and reprobed with anti-MLV antisera (bottom panel). Abbreviations: CA, capsid; +, extracts from 293T cells transfected with the A3A or A3G transgenes.

Several studies, including our own, have shown that A3 proteins inhibit infection by cytidine deaminase-independent means, including inhibiting reverse transcription [47], [58]–[61]. Indeed, we saw no evidence of deamination of either M-MLV or MMTV by endogenous mouse A3 (Table 1) and instead have found that the mouse restriction factor works by inhibiting an early step of reverse transcription [47], [58]. To determine if the A3A or A3G proteins were inhibiting murine retrovirus infection by this mechanism *in vivo*, we isolated virions from M-MLV infected spleens and performed endogenous reverse transcription (enRT) assays. Virions isolated from either of the A3G strains showed diminished RT activity, and the level of activity was proportional to the amount of packaged protein (Figure 5A and 5C). In contrast, virions isolated from either the A3A^high^ or A3A^low^ strains showed RT activity similar to those isolate from A3 knockout mice (Figure 5B). When we examined these particles for the presence of A3A protein, we found that there was none packaged into the virions (Figure 5C). We were unable to perform similar assays with *in vivo* produced MMTV, because the only cell-free virus in mice is found in milk and mammary tumors and we have not yet established breeding colonies of virus-infected human A3 transgenic mice.

### Target cell A3A and A3G restrict infection by incoming viruses

**Figure 6:**
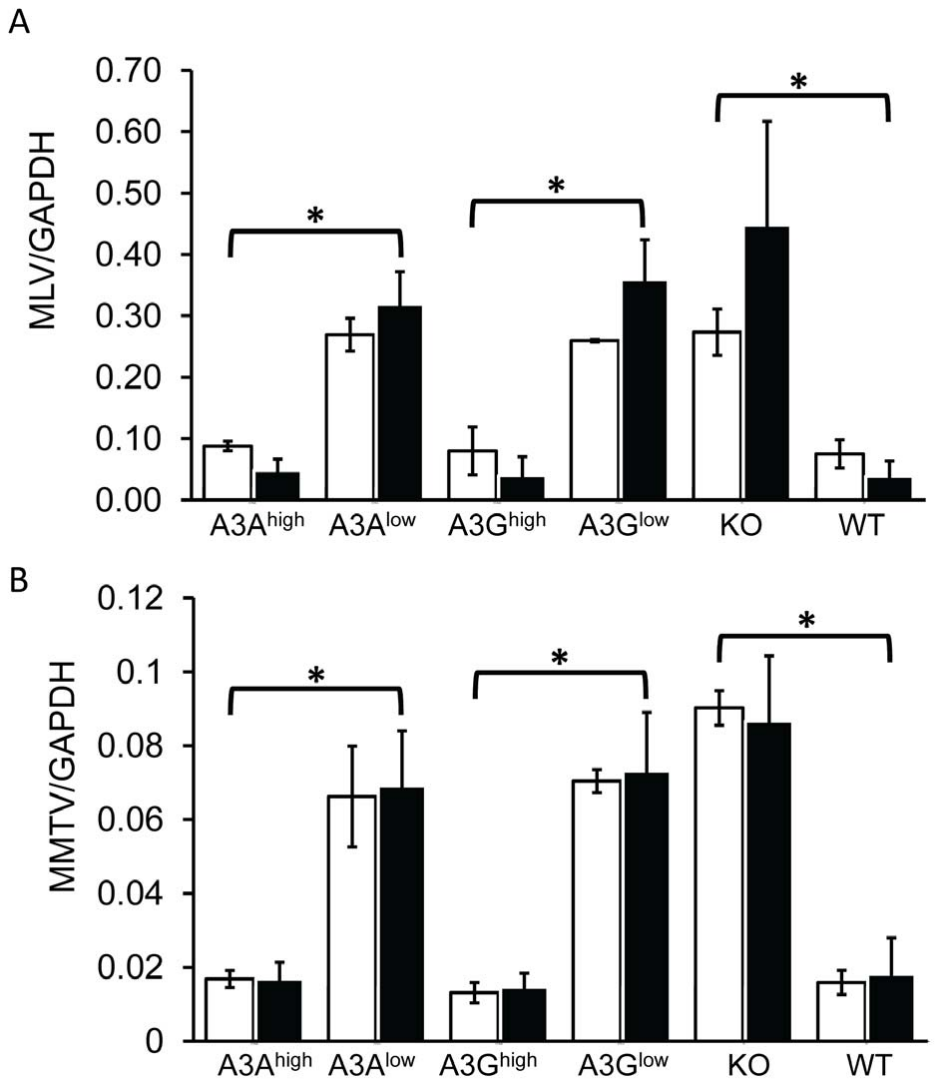
Expression of A3A and A3G in BMDCs restrict incoming murine retroviruses. A) Infection of BMDCs with M-MLV. RT-qPCR analysis of genomic DNA with M-MLV-specific primers, normalized to GAPDH. B) MMTV infection of BMDCs with MMTV. RT-qPCR analysis of genomic DNA with MMTV-specific primers, normalized to GAPDH. Shown are the results of 2 independent experiments (open bars, expt.1; closed bars, expt. 2) with 3 technical replicates in each experiment. Error bars (standard deviation) and p values were calculated for each experiment. *, p≤.01 based on one-way ANOVA.

Another means by which both mouse and human A3 proteins restrict infection is by targeting incoming virus, particularly in sentinel cells of the immune system [22]–[24], [32], [42]. To determine if this mechanism was in effect in the transgenic mice, particularly the A3A transgenic mice which restricted infection without virion incorporation, we isolated BMDCs from the different transgenic mice and infected them with M-MLV or MMTV. Infection of the A3A^high^ and A3G^high^ strains was dramatically reduced to levels seen in wild type C57BL/6 BMDCs at 24 hr post-infection, while infection of BMDCs from the low-expressing A3A and A3G strains was not significantly different than knockout cells (Figure 6A and 6B). The lack of target cell restriction in the A3G^low^ strain may reflect the low level of transgene expression in BMDCs (Figure S2). BMDCs are terminally differentiated cells and therefore are non-replicating; both MLV and MMTV require active cell division for productive infection and thus the effect of A3A was likely on initial infection and not virus spread [62], [63]. Thus, A3A seems to function as a target cell restriction factor, while A3G has the potential to inhibit infection both through virion incorporation and in the target cell.

### Vif overcomes A3G-mediated restriction in vivo

Since A3G is packaged into MLV particles and restricted infection in the same manner as is seen with HIV, we next tested whether the HIV Vif protein would counteract it in the A3G transgenic mice. We introduced the picornavirus 2A peptide into a replication-competent molecular clone of Friend-MLV downstream from *env* (F-MLV-2A) and then engineered *vif* into this construct (F-MLV-2A-vif) (Figure S4) [64]. After transduction of these constructs into NIH3T3 cells, virus was isolated and used to infect 293FT cells (expressing the MLV receptor MCAT-1) transiently transfected with an A3G expression vector. While replication of the parental virus F-MLV-2A was restricted by more than 3 logs in A3G-expressing cells, the F-MLV-2A-vif virus was inhibited by less than 1 log (Figure S4).

**Figure 7:**
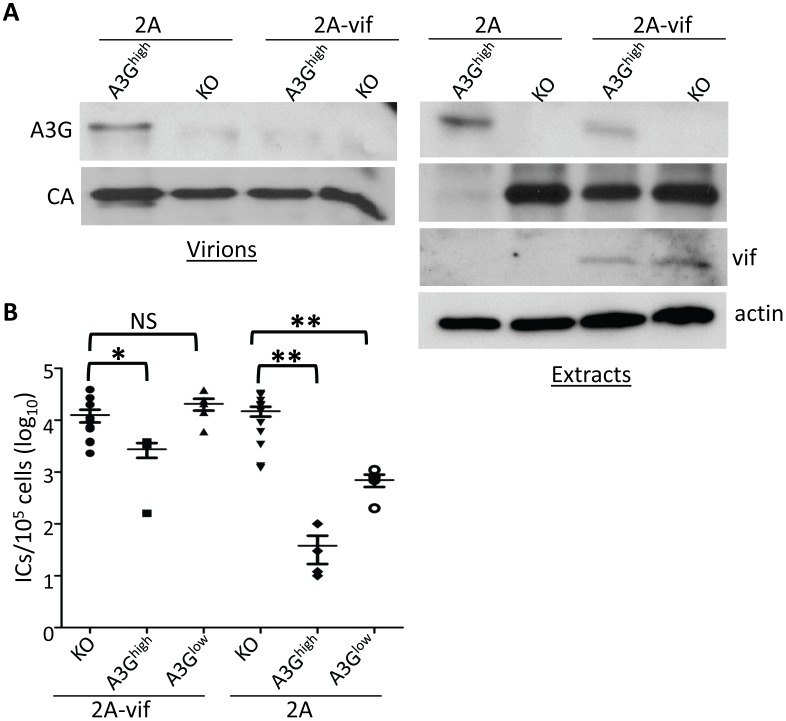
Vif counteracts A3G in transgenic mice. Newborn mice were infected with F-MLV-2A or F-MLV-2A-vif. A) Splenic extracts and isolated virions were analyzed by western blot. A3G was detected with anti-A3G antisera. Shown is a representative western blot from individual mice (KO and A3G^high^) infected with F-MLV-2A and F-MLV-2A-vif. This experiment was repeated twice with 1 additional mouse of each genotype and gave similar results. B) Virus titers in spleens were measured. Each point represents the titer obtained from an individual mouse; the average for each group is shown by a horizontal bar. The transgenic mice were derived from 1–3 litters each; the knockout mice are the littermates of the transgenic mice. N = 11 KO, 4 A3G^high^ and 5 A3G^low^ mice infected with F-MLV-2A-vif and 12 KO, 4 A3G^high^ and 4 A3G^low^ mice infected with FMLV-2A. *, p≤0.02; **, p≤0.004; NS, not significant (Mann-Whitney T test).

To determine if the F-MLV-encoded Vif would also counteract A3G-mediated restriction *in vivo*, we infected newborn pups from the A3G^high^ and A3G^low^ strains with the two viruses and examined *in vivo* expression of Vif, packaging of A3G protein into virions and virus levels in spleen at 16 days post-infection. Vif was readily detected in splenic lysates of F-MLV-2A-vif-infected mice and A3G protein in these lysates was reduced compared to those prepared from F-MLV-2A-infected mice (Figure 7A). Viral RNA from F-MLV-2A and F-MLV-2A-vif virions was normalized by RT-qPCR and equal amount of virus were analyzed by Western blots to determine A3G incorporation. While A3G was efficiently packaged in the parental F-MLV-2A virions, no packaged protein was detected in the F-MLV-2A-vif virus (Figure 7A). Similar to what was seen with M-MLV, replication of the parental F-MLV-2A virus was inhibited by about 3 logs and 1 log in A3G^high^ and A3G^low^ mice, respectively. Indeed, the lower level of CA protein seen in splenic extracts from F-MLV-2A-infected A3G^high^ mice reflects the low titers (Figure 7A). The F-MLV-2A-vif virus was inhibited by less than a log in the A3G^high^ strain, while in the A3G^low^ strain, Vif completely abrogated A3G's inhibition of infection (Figure 7B). In addition, F-MLV-2A was extensively deaminated in the A3G transgenic mice, while deamination of F-MLV-2A-vif was dramatically reduced (Table 1). This was likely due to Vif-mediated inhibition of A3G packaging into virions.

## Discussion

A3 family members play important roles in the host's defense against viral infections, in particular against retroviruses. While much is known about the function of human A3 proteins *in vitro*, less is known about their function *in vivo*. In contrast, studies in A3 knock-out mice infected with various murine retroviruses have begun to elucidate the role of these proteins during *in vivo* infection [32], [33], [41]. Here we took advantage of mA3 knockout mice and generated transgenic mice that express human A3G and A3A in the absence of mouse A3, with the goal of developing a system with which to study the *in vivo* effects of individual human proteins. Primates express 7 A3 proteins, many with overlapping target motif sites. Due to the common target motif sites, it is difficult to fully discriminate the contribution of each A3 protein to inhibiting retrovirus infection *in vivo* or in primary cells. The development of transgenic mice that express only one A3 protein allows us to delineate the function of each A3 protein *in vivo* without the interference of the other A3 proteins.

An additional limitation of previous studies done on human A3 proteins is the reliance on transfecting constructs expressing A3 proteins, which may not reflect the endogenous levels of a protein expression found *in vivo*. The transgenic mice described here express A3A or A3G proteins at levels similar to those seen in human cell in the absence of other A3 proteins, thus providing a model for better understanding their individual action in the context of virus infection.

We initiated these studies by creating transgenic mice with human *A3* genes with very different properties: A3G, which is a potent inhibitor of HIV-Vif infection, has two CDA domains and is cytoplasmic, and A3A, whose role in HIV inhibition is less-well characterized, has a single CDA domain, is not degraded by Vif and is believed to be both nuclear and cytoplasmic. Additionally, A3A preferentially deaminates TC motifs [26], while A3G preferentially deaminates at CC motifs [7], [8]. We used a β-actin promoter to drive these transgenes, to give us the ability to compare the function of the A3 proteins in different tissues and cell types. Two A3A and A3G mouse strains each were generated, expressing levels of these proteins within the range or at levels lower than that seen in human cells. This likely has relevance to what occurs in individual humans, where non-coding region polymorphisms in A3 genes alter expression levels and may influence progression to HIV-induced disease [65]–[67]. We were thus able to functionally demonstrate the dose-dependent effects of both A3A and A3G on infection by two different murine retroviruses, MLV and MMTV, in an *in vivo* model.

A number of studies have suggested that A3G and A3A inhibit retrovirus infection by different means. A3G largely functions as a CDA during reverse transcription after packaging into virions, while A3A seems to work in target cells to block incoming virus infection, either via deaminase-dependent or –independent means [15], [23], [24]. We were able to examine the mode of restriction used by A3A and A3G in restricting murine retroviruses *in vivo*, in a system where only a single human A3 gene was expressed. Our findings showed that A3G was packaged inside MLV virions *in vivo* and that it inhibited infection by MMTV and MLV primarily by cytidine deamination of viral DNA. On the other hand, A3A was not packaged inside MLV virions, did not hypermutate the MLV genome and had no effect on viral reverse transcription in particles. However, both A3A and A3G expressed in BMDCs derived from the transgenic mice inhibited early reverse transcription by both incoming MLV and MMTV. These data support previous *in vitro* studies suggesting that A3A predominantly functions in target cells to inhibit infection by both deamination-dependent and –independent means [23], [24].

Interestingly, while we saw no evidence of deamination of MLV by A3A, MMTV showed a low level of deamination of proviral DNA at the signature deamination motif used by A3A and MMTV infection was reduced to a greater extent than M-MLV in the A3A^high^ mouse strain. Indeed, unlike M-MLV, MMTV infection was inhibited similarly in the A3A^high^ and A3G^high^ strains (Figure 3B). The discrepancy in deamination between MLV and MMTV could be due to the fact that MLV has fewer target motif sites for A3A compared to MMTV (33 GA motifs in M-MLV vs. 56 GA motifs MMTV in the sequenced regions). It is also possible that A3A is packaged into MMTV virions or that A3A interacts differently with the MMTV reverse transcription complex. Finally, these viruses may use different target cells of infection *in vivo* that express different levels of A3A. Nonetheless, the extent of MMTV deamination in the A3A^high^ vs. A3G^high^ mice, which have equivalent transgene expression in lymphoid tissues, was significantly lower (6/52 genomes vs. 32/56 genomes, respectively; p≤0.0001 Fisher's test), suggesting that both CDA-dependent and –independent mechanisms are important for MMTV restriction.

It has been suggested that a major role of A3 proteins is to prevent zoonoses [28], [30], [40]. Several human A3 proteins have been shown previously to restrict murine retroviruses *in vitro*, at least in human cell lines [68], [69]. Here we show that indeed A3A and A3G potently restrict the infection of the murine retroviruses MLV and MMTV when they are the only human proteins expressed *in vivo.* Indeed, although we previously demonstrated that endogenous mouse A3 restricts M-MLV and MMTV *in vivo* [32], [33], in the short-term *in vivo* infection assay used here, both the A3A and A3G transgenes were more effective at inhibiting infection than the mouse protein, even when expressed at much lower levels (Figure 3). Our findings thus support the *in vitro* studies suggesting that A3 proteins may act as means of prevention of zoonoses.

We also showed that Vif can effectively counteract A3G in this *in vivo* model, demonstrating that the machinery needed for Vif-mediated A3G degradation does not show species-specificity. While much is known about A3G-Vif interactions from *in vitro* studies [70], [71], our F-MLV-Vif/A3G model has the potential to greatly increase our understanding of Vif-A3G functions *in vivo*. Previous studies using humanized mouse models have also studied A3-Vif interactions *in vivo* and shown that HIV proviral DNA is deaminated, even in viruses containing *vif* [72]. We also found that the presence of *vif* in F-MLV diminished A3G deamination and that this was proportional to the level of transgene expression (Table 1). The advantage of our model is that different A3 proteins can be studied in isolation in the mice and thus, the interaction of Vif with individual A3 proteins can be distinguished. This model could also be used to test small molecule inhibitors of A3G/Vif interactions *in vivo*.

Other viruses that are thought to be inhibited by human A3 proteins such as parvoviruses, herpes simplex I virus, and hepatitis B virus could also be studied in these mice [20], [34], [36], [39], [73]. In addition, A3 proteins have been linked to DNA damage and genomic mutations and it has been suggested that A3A deaminates viral, nuclear, and mitochondrial DNA [74], [75]. However, most of this work has been done using immortalized cell lines or tissue samples from patients and thus cannot fully recapitulate the role of A3 proteins in DNA damage and genomic mutations. These newly developed animal models allow for a closer study of the role the A3 proteins on genomic integrity.

In summary, our A3 transgenic models have the potential to increase our understanding of how the human proteins function to restrict virus infection *in vivo*. These mice should be useful for the development of therapeutics that enhance A3 proteins' antiviral function and to better define the deleterious effects these proteins might have on the host.

## Materials and Methods

### Ethical statement

All mice were housed according to the policies of the Institutional Animal Care and Use Committee of the University of Pennsylvania and all studies were performed in accordance with the recommendations in the Guide for the Care and Use of Laboratory Animals of the National Institutes of Health. The experiments performed with mice in this study were approved by this committee (IACUC protocol #801594). Human studies were approved by the Institutional Research Board of the University of Pennsylvania (IRB protocol #81703). Informed consent was not obtained as all human samples were decoded and analyzed anonymously.

### Transgene construction

The A3A and A3G constructs were kindly given to us by Matija Peterlin [22]. The A3A and A3G fragments from these constructs were subcloned into pcDNA3.1(−) myc/his (Invitrogen) and then into the pCAGGs vector (courtesy of Yongwon Choi). Construction details are available upon request. All clones were sequenced to verify the inserts.

### Generation of mice

The pCAGGs vector carrying myc-tagged A3A or A3G genes was cut with SalI and HindIII to remove vector sequences and the insert was gel-purified prior to micro-injection. Fertilized eggs of C57BL/6 mice were injected with purified DNA fragments by the Transgenic and Chimeric Mouse Facility of the University of Pennsylvania. The transgenic mice were then backcrossed with mouse A3 knockout mice to generate animals containing functional copies of only the human genes. All transgenic lines were maintained by breeding with mA3 knockout mice, so the transgenes were carried in heterozygotes. This allowed us to generate non-transgenic, matched controls for infection studies. Mice were genotyped for A3A using primers 5′-ATGGCATTGGAAGGCATAAG-3′/5′-CAAAGAAGGAACCAGGTCCA-3′ and A3G using primers 5′-GGGACCCAGATTACCAGGAG-3′/5′-GCAGATTATTCCAAGGCTCAA-3′, and for the mouse A3 gene, as previously described [33].

### Transgene RNA analysis

Tissues were harvested from 3 month old mice and RNA was isolated with the use of Trizol© (Invitrogen). RNA was further processed for the elimination of any residual phenol according to the RNA cleanup method per the manufacturer's recommendation (Qiagen) and then treated with DNaseI to eliminate any contaminating genomic DNA (Qiagen). cDNA was produced from the purified RNA using the SuperScript III First Strand Synthesis System for RT-PCR (Invitrogen). RT-PCR was performed using the Power SYBR Green PCR master mix kit (Applied Biosystems) with the same A3A and A3G primers described above. For the transcriptional profile, a standard curve was made using known quantities of plasmid DNA and used to calculate the absolute copy numbers for A3A, A3G and GAPDH. The copy number for A3A and A3G were then normalized according to the levels of GAPDH.

### Western blots

Cells and whole tissues were lysed for 30 min. on ice in protein lysis buffer (50 mM Tris pH 7.4, 150 mM NaCl, 1 mM EDTA, 1% Triton-X-100, 1% deoxycholate, 0.1% SDS and protease inhibitor cocktail) and then were sonicated and centrifuged at 10000 g for 10 minutes. To quantify the levels of the A3A and A3G transgene expression, 250 µg and 100 µg of each tissue extract, respectively, were analyzed on 10% SDS polyacrylamide gels. The following antibodies were used: polyclonal goat anti-MLV antibody (NCI Repository), mouse anti-β-actin antibody (Sigma Aldrich), rabbit anti-GAPDH (Cell Signaling Technology), rabbit anti-vif (NIH AIDS Reference and Reagent Program), rabbit anti-myc (Roche) and rabbit anti-A3G (cem15 c29; NIH AIDS Reference and Reagent Program) which recognizes both A3G and A3A. HRP-conjugated anti-rabbit (Cell Signaling Technology), anti-goat and anti-mouse antibodies (Sigma Aldrich) were used for detection, using either ECL kits (GE Healthcare Life Sciences) or the Supersignal West Femto Chemiluminescent substrate (Thermo Scientific). For western blot analysis of virions, viral RNA was quantified by RT-qPCR, as previously described [58] and equal amounts were loaded on gels.

### Deamination assay

Splenocytes were isolated from the transgenic mice and stored at -80°C until use. As controls, 293T cells transfected with A3A- or A3G- expressing [22] were also used. Cytoplasmic extracts were prepared in lysis buffer (10 mM Hepes pH 7.3, 10 mM NaCl, 1.5 mM MgCl_2_, 0.5 mM PMSF, 1 mM DTT and 10 µM ZnCl_2_) using 200 µL lysis buffer/10^7^ cells, incubated on ice for 15 min and then repeatedly passed through a 29 gauge needle. The supernatants after centrifugation (800xg for 5 min) were dialyzed (20 mM HEPES-OH, 100 mM NaCl, 2 mM EDTA, 1 mM DTT, 0.5 mM PMSF and 5% glycerol pH 7.3 @4°C) and subsequently quantified by Bradford assay. The assay was performed with 3′-fluorescein labeled 50-mer DNA substrates (S50-XXC; ATT ATT ATT ATT XXC ATT TAT TTA TTT ATT TAT GGT GTT TGG TGT GGT T –FAM; IDT) that contained a cytosine at position 15 in different sequence context, S50-TTC, S50-CCC, S50-AGC. OneµM of substrate was incubated with extract (37.5 µg/µL final, 5% dialysis buffer), 0.05 units/µL UDG (NEB) and 0.1 µg/µL RNaseA in 20 mM Tris, pH 8, 1 mM DTT, 1 mM EDTA. After 2.5 hrs the reaction was quenched with SDS (1% final) and frozen. The reactions were extracted with phenol/chloroform/isoamyl alcohol and then chloroform. Abasic sites were cleaved with NaOH (150 mM final) and heating to 95°C for 10 min. Samples were mixed with equal volume of formamide and loading dye, run on 20% 1XTBE/7M Urea polyacrylamide gel, and imaged using a Typhoon variable model imager.

### Primary cell isolation

BMDCs were generated from the A3A, A3G transgenic mice as well as the wild type and mA3 knockout mice as previously described [76]. The cells were differentiated with recombinant murine granulocyte-macrophage colony-stimulating factor (20 ng/ml; Invitrogen). Macrophages, B and T cells were obtained from the peripheral blood of A3A and A3G transgenic, C57BL/6 and A3 knockout mice of similar ages. The blood was treated with ACK (Ammonium-Chloride-Potassium) lysis buffer to remove red blood cells. After washing with PBS/2% FCS, the cells were incubated with anti-CD3-APC, anti-CD19-PE and anti-CD11b-PercP for 30 minutes on ice. Stained cells were washed 2 times with PBS/2% FBS and sorted using a BD FACSCalibur cell sorter and CellQuest Pro software. The sorted cells were lysed for RNA isolation using the RNeasy kit (Qiagen) according to the manufacturer's instructions.

### Virus isolation

M-MLV was isolated from NIH 3T3 fibroblasts stably infected with virus as previously described [77]. F-MLV-2A and F-MLV-2A-vif were isolated from Mus Dunni cells. M-MLV and F-MLV were titered as described in the next section. F-MLV-2A was constructed from an F-MLV vector (pLRB302) provided by Mario Santiago into which the 2A peptide sequence designed for the construction of multicistronic vectors was inserted [64], [78] (Figure S4 legend). For virus isolation from spleen, splenocytes were collected and incubated in RPMI 1640, 10% FCS, nonessential amino acids, and penicillin/streptomycin for 48 h. The media was passed through a 0.4-µm filter, treated with 20 U/ml DNase I (Roche) at 37°C for 30 min, and pelleted through a 30% sucrose cushion. After resuspension, M-MLV was titered on NIH 3T3 cells and F-MLV on Mus Dunni cells, as well as quantified by reverse-transcribed RT-qPCR and analyzed on Western blots with anti-MLV antisera. The primers used for virus quantification were located in the *env* genes; (M-MLV: 5′-CCTACTACGAAGGGGTTG-3′/5′-CACATGGTACCTGTAGGGGC-3′; F-MLV 5′-TACAGGGAGCTTACCAGGCA-3′/5′-GTTCCTATGCAGAGTCCCCG-3′). MMTV(RIII) virus was isolated as previously described from tumors of A3 knockout mice [79]. Real-time PCR was used to calculate approximate MMTV virion RNA levels in the preparation, as previously described [47].

### Infectivity assays

M-MLV infection levels in the spleens of the infected mice were determined by infectious center (IC) assays using a focal immunofluorescence assay, as previously described [32]. F-MLV-2A and FMLV-2Avif titers were performed as follows: Mus Dunni cells were seeded on a 12-well plate at a concentration of 10^5^ cells per well. The cells were co-cultured the next day with splenocytes from infected mice at 10-fold dilutions. At 2 hours post-infection 2 ml of media was added in each well and the cells were incubated at 37°C with 5% CO_2_ for 48 hrs. The cells were fixed with 100% ice-cold methanol for 10 minutes and then incubated with an anti-FMLV env antibody for 1hr at 4°C followed by anti-mouse Alexafluor488 antibody (Invitrogen) for 1hr at 4°C. ICs were counted using a Nikon Diaphot 300 fluorescence microscope.

### *In vivo* infections

For all experiments, newborn mice were generated by crossing mice heterozygous for the transgene, homozygous for mouse A3 knockout with A3 knockout mice, generating both transgenic and nontransgenic mouse A3 knockout controls. Infections were done without genotyping the mice; genotyping was performed at 10 days post-infection. C57BL/6 mice were bred separately for these experiments. For MLV infections, two day old mice were infected by IP injection of 10^5^ ICs M-MLV or 10^3^ ICs FMLV-2A and FMLV-2A-vif, and harvested 16 days post-infection, as previously described [58]. For MMTV(RIII) infections, 1x10^6^ virions were injected IP into 5 day old mice. Infected mouse spleens were harvested 3 weeks post-infection. To measure the amounts of viral DNA in the spleens of the infected mice, splenic DNA was isolated using the DNeasy Blood and Tissue Kit (Qiagen). RT-PCR was performed using the Power SYBR Green PCR master mix kit (Applied Biosystems). For MLV, the *env* primers described in the Virus Isolation section were used. The primers used for MMTV detection were 5′-CGTGAAAGACTCGCCAGAGCTA-3′/5′-GAAGATGATCTTCAAGGGCAATGCCT-3′. GAPDH primers were used in both cases for normalization 5′-CCCCTTCATTGACCTCAACTACA-3/5′-CGCTCCTGGAGGATGGTGAT-3′.

### eNRT assays

Equal amounts of virus (normalized by RNA levels as described in the preceding section) isolated from the splenocytes of M-MLV infected A3A, A3G, A3 knockout and BL/6 mice were incubated in EnRT buffer (1× PBS, 2.5 mM MgCl_2_, 0.01% Nonidet P-40, 1 mM dNTPs) at 37 °C. Fractions of the reactions were removed at 0, 30 min, 1 h, 2 h, and 4 h and added to 40 µg of sonicated salmon sperm DNA. DNA was isolated from fractions using the Qiagen DNeasy Blood and Tissue Kit (Qiagen). PCR was performed using MLV strong stop primers to measure the level of early reverse transcripts: F primer, 5′-5′-CCTCCGATTGACTGAGTCGCCCC-3′; R primer, 5′-ATGAAAGACCCCCGCTGACGG-3′.

### Sequencing

DNA from the spleens of MLV- and MMTV-infected A3A, A3G, BL/6 and A3 knockout mice was isolated as described above. RNA was also isolated from M-MLV virions produced by the infected splenocytes of the A3G transgenic mice using the Rneasy Mini kit (Qiagen) and cDNA was then produced using the SuperScript III First Strand Synthesis System for RT-PCR (Invitrogen). A 549bp region from the M-MLV envelope was amplified using the primers 5′-CCAATGGAGATCGGGAGACG-3′/5′-GTGGTCCAGGAAGTAACCCG-3′, a 586bp region from the F-MLV envelope was amplified using the primers 5′-AGCCCTCACCAGGTCTACAA-3′/5′-ATGAGGTGACCTGTTTCCCG-3′ and a 673bp region from the MMTV(RIII) LTR was amplified by nested PCR using primers 5′-GAAGATCTTCCCGAGAGTGTCCTACAC-3′/5′-GAAGATGATCTTCAAGGGCAATGCCT-3′ for the 1^st^ round and a second forward primer 5′-AATTCGGAGAACTCGACCTTCC-3′ with the same reverse primer for the 2^nd^ round of amplification. Bands were excised and the fragments were cloned into pCR2.1-TOPO vector as specified by the manufacturer (Invitrogen). Sequences were aligned using the ClustalW program, and G-to-A mutations were annotated by Hypermut (www.hiv.lanl.gov/content/sequence/HYPERMUT/hypermut.html).

### Target cell assays

BMDCs were infected by the spinoculation method as previously described [56]. Briefly, BMDCs were plated in a 96-well plate at a cell density of 10^5^/100 µl. MMTV RIII (3×10^7^ virions/10^5^ cells) or M-MLV (MOI = 1) was added to the cells and the plates were centrifuged at 1,200 × *g* for 120 min at room temperature. After centrifugation, the cells were incubated for 24hrs at 37°C. DNA was extracted using the Dneasy kit (Qiagen) according to the manufacturer's instructions. RT-qPCR was performed to examine the infection levels, as described above (*In vivo* infections).

### Statistical analysis

Statistical analysis was performed using GraphPad/PRIZM software. Tests used to determine significance are described in the figure legends.

## Supporting Information

Figure S1 A) Western blots of tissues from A3G^high^ and A3G^low^ strains, probed with anti-A3G and anti-GAPDH antibodies. B) Western blots of tissues from A3A^high^ and A3A^low^ strains, probed with anti-A3G antibody that also recognizes A3A (cem15 C-29) and anti-GAPDH antibodies. The mice used in this analysis were uninfected.
(PDF)

Figure S2 Transgene expression in hematopoietic lineage cells. T cells, B cells, macrophages (M) and bone marrow-derived dendritic cells (DC) were purified from the mice of each genotype as described in the text and RNA isolated from the purified cells was analyzed by RT-qPCR for transgene expression. Shown are the averages for 3 (T cells, B cells and macrophages) or 2 (BMDCs) different mice. This experiment was performed twice with similar results; shown is a representative experiment. Error bars denote standard deviation.
(PDF)

Figure S3 Deamination of MMTV viral DNA in A3A and A3G transgenic mice. A) Splenic DNA was isolated from the MMTV-infected mice described in Fig. 4 and cloned and sequenced. In most cases > 10 sequences from 3-4 different mice were analyzed, as indicated in the figure. Shown are the G to A changes in the sequences; other mutations are indicated in Table 1. Red  =  GG > AG, cyan  = GA > AA, green  = GC > AC and magenta  = GT > AT transitions. Red arrows denote mutation hotspots seen in viruses isolated from A3G^high^ and A3G^low^ mice; black arrows denote hotspots identified only in A3G^high^ mice.
(PDF)

Figure S4 Construction of FMLV-2A-vif. **Upper panel:** A plasmid encoding a full-length replication-competent clone of B-tropic F-MLV was modified by adding a 2A peptide sequence from picornavirus (P2A) in frame with the C terminus of the envelope gene, followed by a Not1 restriction site and a stop codon. The resulting plasmid is named FMLV-2A. The *vif* gene from NL4-3 was then amplified by PCR and clone in frame into the Not1 site to generate FMLV-2A-vif. **Lower panel:** 293FT cells were transfected with 1ug of FMLV-2A or FMLV-2A-vif alone or in combination with 50ng hA3G or empty vector (pcDNA). At two days post transfection, the supernatant was harvested and assayed for the presence of infectious FMLV by plating on *Mus dunni* cells by an IC assay. The data are representative of two independent experiments.
(PDF)

Table S1 Deamination hotspots in A3G transgenic mice. Each of the hotspots appeared once in the sequenced region. Shown is the fraction of sequences containing the G to A change. There were no deamination hotspots for either virus in the A3A transgenic mice.
(PDF)
