# Supplementary material for: Different Modes of Retrovirus Restriction by Human APOBEC3A and APOBEC3G In Vivo
Source: PLoS Pathog. 2014 May 22;10(5):e1004145. doi: 10.1371/journal.ppat.1004145 (PMC4031197; doi:10.1371/journal.ppat.1004145)
Supplement: Figure S1 — A) Western blots of tissues from A3Ghigh and A3Glow strains, probed with anti-A3G and anti-GAPDH antibodies. B) Western blots of tissues from A3Ahigh and A3Alow strains, probed with anti-A3G antibody that also recognizes A3A (cem15 C-29) and anti-GAPDH antibodies. The mice used in this analysis were uninfected. (PDF) [file ppat.1004145.s001.pdf]

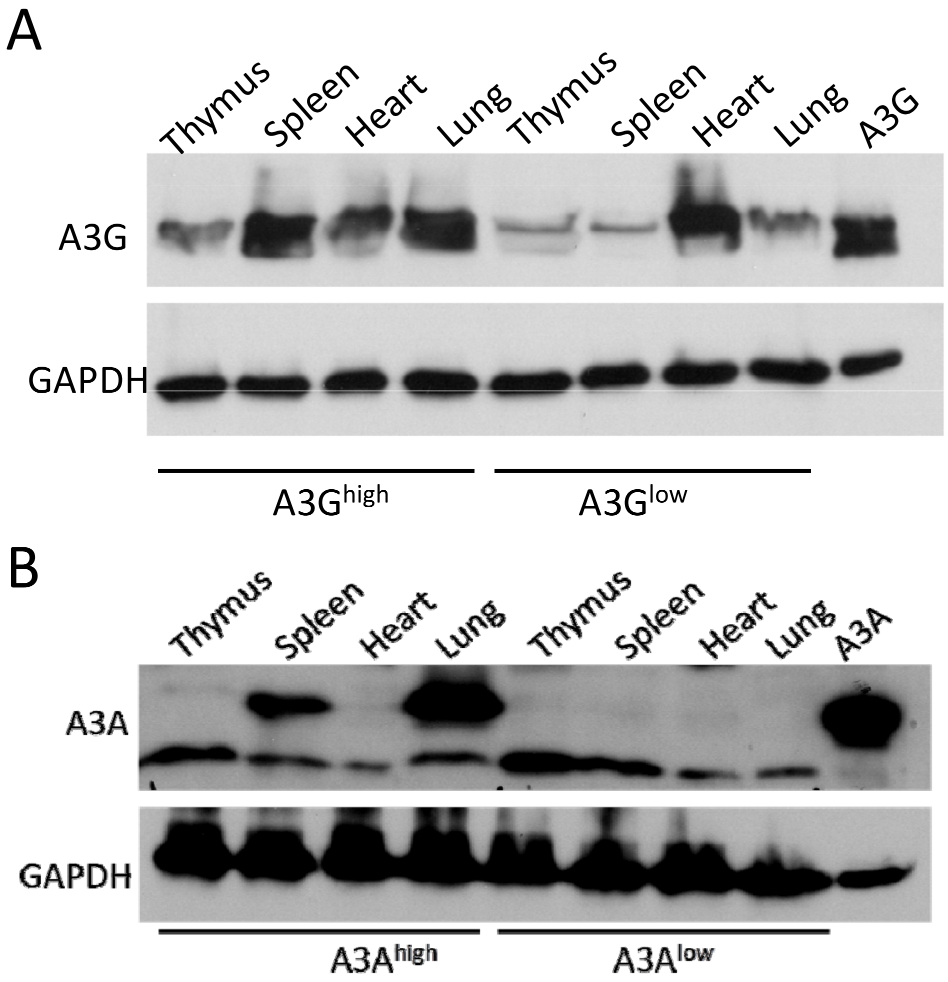

Figure S1. A) Western blots of tissues from A3G^{high} and A3G^{low} strains, probed with anti-A3G and anti-GAPDH antibodies. B) Western blots of tissues from A3A^{high} and A3A^{low} strains, probed with anti-A3G antibody that also recognizes A3A (cem15 C-29) and anti-GAPDH antibodies. The mice used in this analysis were uninfected.
