# Supplementary material for: Different Modes of Retrovirus Restriction by Human APOBEC3A and APOBEC3G In Vivo
Source: PLoS Pathog. 2014 May 22;10(5):e1004145. doi: 10.1371/journal.ppat.1004145 (PMC4031197; doi:10.1371/journal.ppat.1004145)
Supplement: Figure S2 — Transgene expression in hematopoietic lineage cells. T cells, B cells, macrophages (M) and bone marrow-derived dendritic cells (DC) were purified from the mice of each genotype as described in the text and RNA isolated from the purified cells was analyzed by RT-qPCR for transgene expression. Shown are the averages for 3 (T cells, B cells and macrophages) or 2 (BMDCs) different mice. This experiment was performed twice with similar results; shown is a representative experiment. Error bars denote standard deviation. (PDF) [file ppat.1004145.s002.pdf]

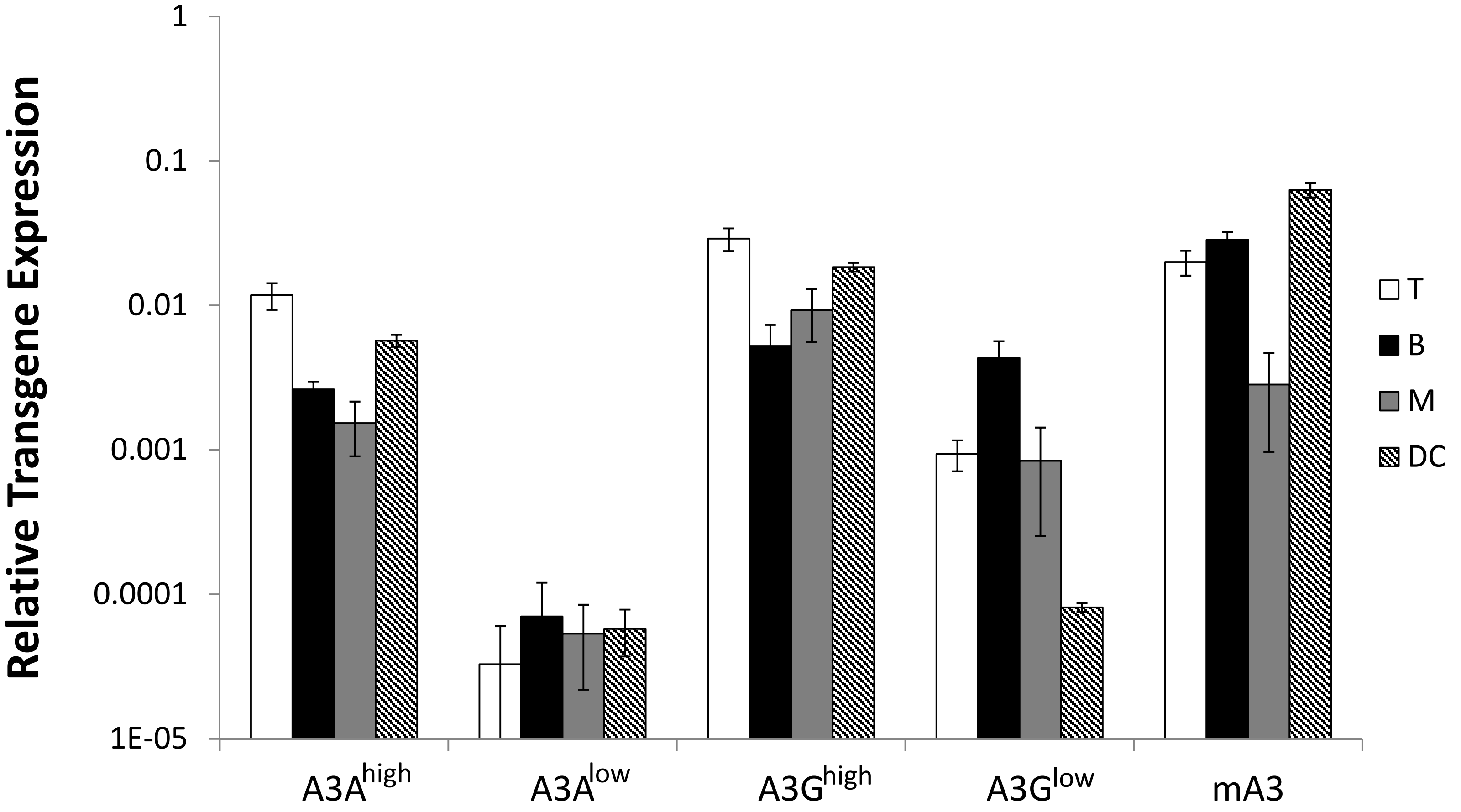

Figure S2. Transgene expression in hematopoietic lineage cells. T cells, B cells, macrophages (M) and bone marrow-derived dendritic cells (DC) were purified from the mice of each genotype as described in the text and RNA isolated from the purified cells was analyzed by RT-qPCR for transgene expression. Shown are the averages for 3 (T cells, B cells and macrophages) or 2 (BMDCs) different mice. This experiment was performed twice with similar results; shown is a representative experiment. Error bars denote standard deviation.
