# Supplementary figures and images for: Different Modes of Retrovirus Restriction by Human APOBEC3A and APOBEC3G In Vivo
Source: PLoS Pathog. 2014 May 22;10(5):e1004145. doi: 10.1371/journal.ppat.1004145 (PMC4031197; doi:10.1371/journal.ppat.1004145)

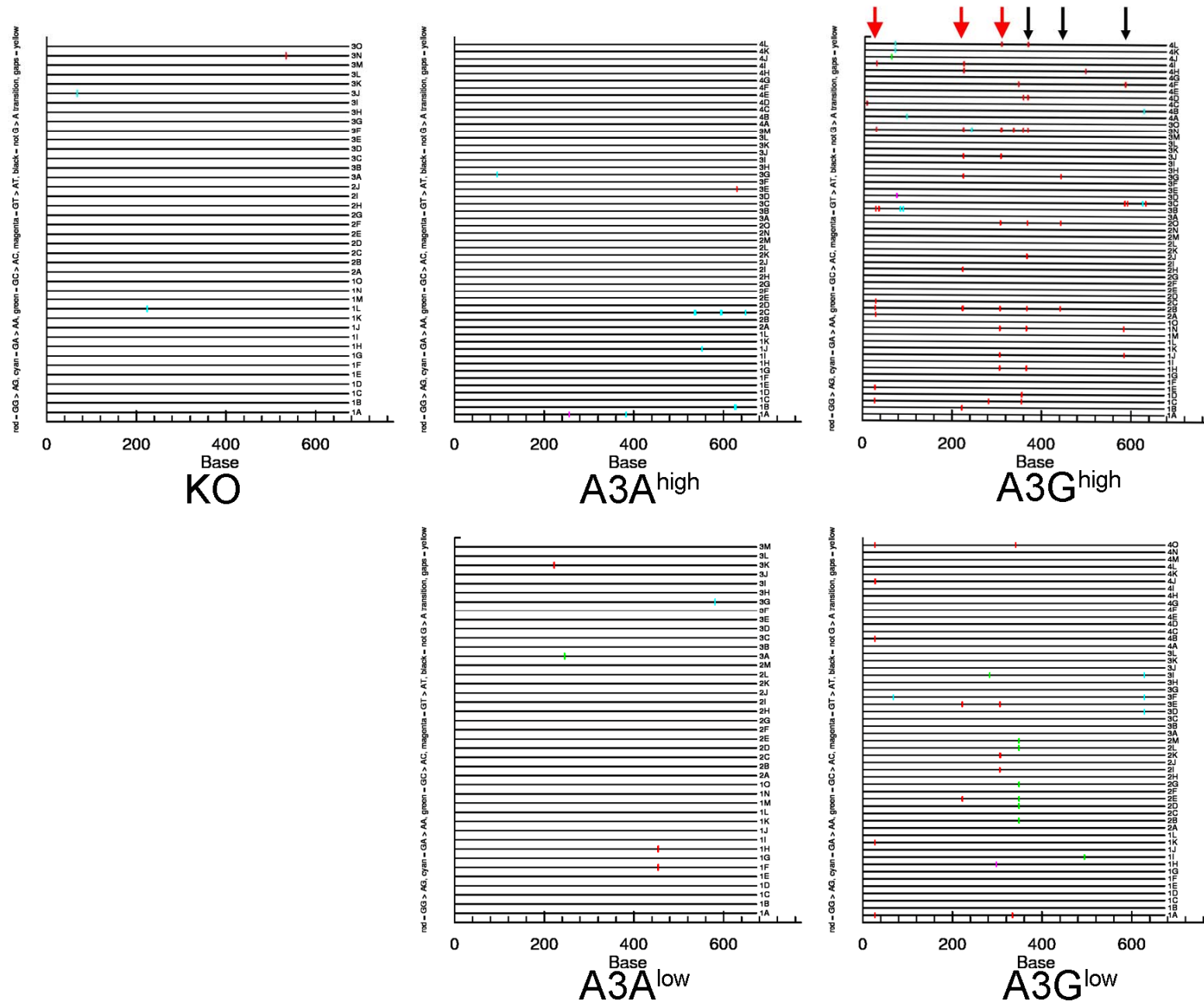

**Figure S3.** Deamination of MMTV viral DNA in A3A and A3G transgenic mice.

Supplement: Figure S3 — Deamination of MMTV viral DNA in A3A and A3G transgenic mice. A) Splenic DNA was isolated from the MMTV-infected mice described in Fig. 4 and cloned and sequenced. In most cases > 10 sequences from 3-4 different mice were analyzed, as indicated in the figure. Shown are the G to A changes in the sequences; other mutations are indicated in Table 1. Red = GG > AG, cyan = GA > AA, green = GC > AC and magenta = GT > AT transitions. Red arrows denote mutation hotspots seen in viruses isolated from A3Ghigh and A3Glow mice; black arrows denote hotspots identified only in A3Ghigh mice. (PDF) [file ppat.1004145.s003.pdf]
