# Supplementary material for: Different Modes of Retrovirus Restriction by Human APOBEC3A and APOBEC3G In Vivo
Source: PLoS Pathog. 2014 May 22;10(5):e1004145. doi: 10.1371/journal.ppat.1004145 (PMC4031197; doi:10.1371/journal.ppat.1004145)
Supplement: Figure S4 — Construction of FMLV-2A-vif. Upper panel: A plasmid encoding a full-length replication-competent clone of B-tropic F-MLV was modified by adding a 2A peptide sequence from picornavirus (P2A) in frame with the C terminus of the envelope gene, followed by a Not1 restriction site and a stop codon. The resulting plasmid is named FMLV-2A. The vif gene from NL4-3 was then amplified by PCR and clone in frame into the Not1 site to generate FMLV-2A-vif. Lower panel: 293FT cells were transfected with 1ug of FMLV-2A or FMLV-2A-vif alone or in combination with 50ng hA3G or empty vector (pcDNA). At two days post transfection, the supernatant was harvested and assayed for the presence of infectious FMLV by plating on Mus dunni cells by an IC assay. The data are representative of two independent experiments. (PDF) [file ppat.1004145.s004.pdf]

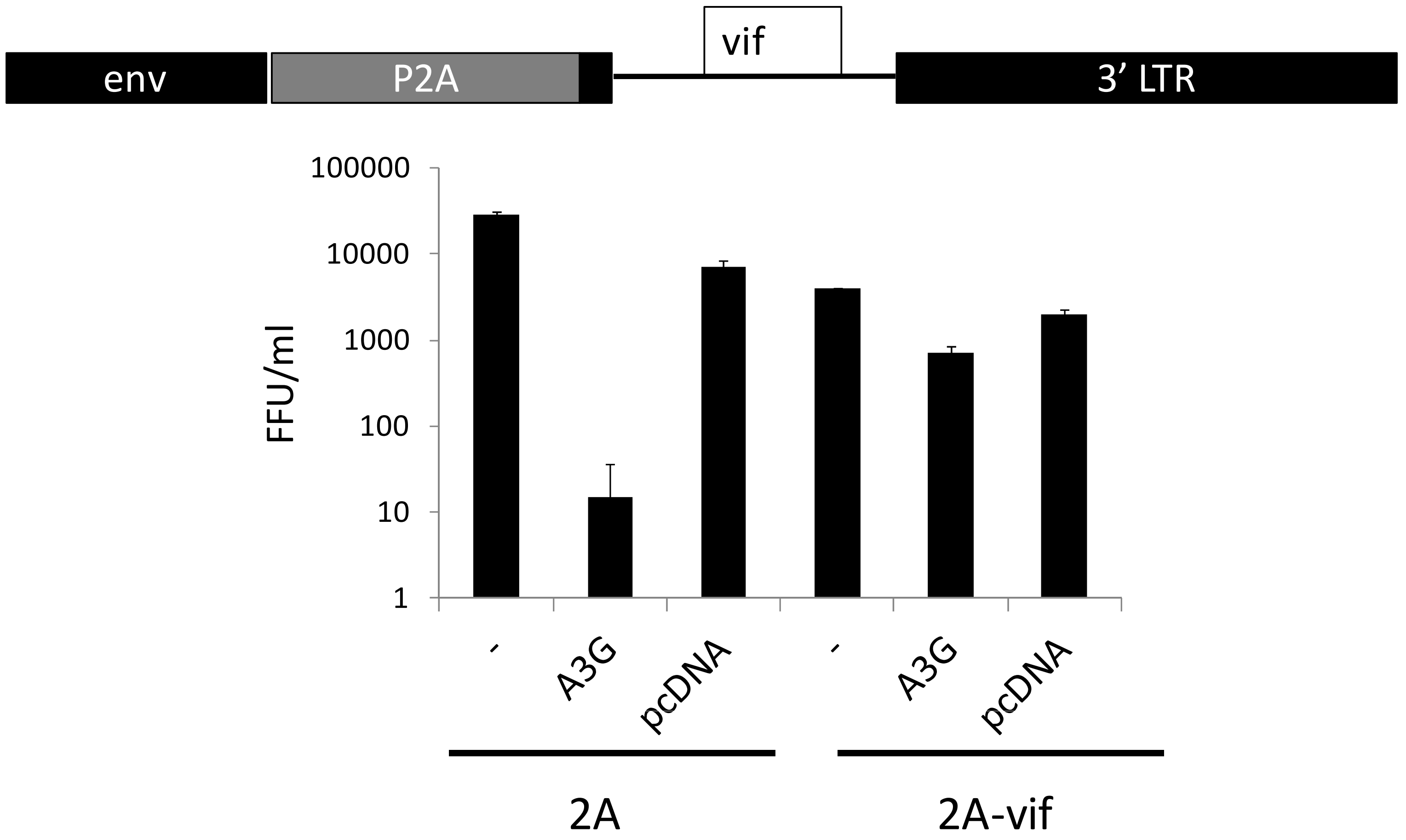

Figure S4. Construction of FMLV-2A-vif. Upper panel: A plasmid encoding a full-length replication-competent clone of B-tropic F-MLV was modified by adding a 2A peptide sequence from picornavirus (P2A) in frame with the C terminus of the envelope gene, followed by a Not1 restriction site and a stop codon. The resulting plasmid is named FMLV-2A. The *vif* gene from NL4-3 was then amplified by PCR and clone in frame into the Not1 site to generate FMLV-2A-vif.

Lower panel: 293FT cells were transfected with 1ug of FMLV-2A or FMLV-2A-vif alone or in combination with 50ng hA3G or empty vector (pcDNA). At two days post transfection, the supernatant was harvested and assayed for the presence of infectious FMLV by plating on *Mus dunni* cells by an IC assay. The data are representative of two independent experiments.
