# Supplementary material for: Different Modes of Retrovirus Restriction by Human APOBEC3A and APOBEC3G In Vivo
Source: PLoS Pathog. 2014 May 22;10(5):e1004145. doi: 10.1371/journal.ppat.1004145 (PMC4031197; doi:10.1371/journal.ppat.1004145)
Supplement: Table S1 — Deamination hotspots in A3G transgenic mice. Each of the hotspots appeared once in the sequenced region. Shown is the fraction of sequences containing the G to A change. There were no deamination hotspots for either virus in the A3A transgenic mice. (PDF) [file ppat.1004145.s005.pdf]

Table S1. Deamination hotspots in A3G transgenic mice. Each of the hotspots appeared once in the sequenced region. Shown is the fraction of sequences containing the G to A change. There were no deamination hotspots for either virus in the A3A transgenic mice.

**SEQUENCE**	**$A3G^{high}$**		**$A3G^{low}$**	
	DNA	RNA	DNA	RNA
**M-MLV**				
TATGGGC	0.20	0.06	0	0
TATTGGG	0.30	0.06	0.21	0
CTCAGGG	0.13	0.06	0.15	0
ATGAGGG	0.23	0.09	0	0
CCCCGGG	0.10	0	0.06	0
ATGTGGG	0.28	0.01	0.11	0.05
TATTGGG	0.35	0.11	0.04	0.03
TCATGGG	0.25	0.045	0.02	0
CGCCGGG	0.10	0	0	0
**MMTV**				
CCTGGGG	0.14		0.08	
AAATGGG	0.14		0.04	
AAAAGGG	0.11		0.06	
ACCAGGG	0.07		0.02	
TATAGGG	0.11		0	
AGGTGGG	0.05		0	
TCGAGGG	0.07		0	
